# Cerebral hemodynamics in children with sickle cell disease in India: An observational cohort study

**DOI:** 10.1097/MD.0000000000029882

**Published:** 2022-07-08

**Authors:** Bhakti Gajjar, Sanjay Sharma, Erum Khan, Pranita Sharma, Pawan Jain, Vikas Goel, Arvind Neral, Jyotish Patel, Mamta Parmar, Kanika Sharma, Vijay K. Sharma, Arvind K. Sharma

**Affiliations:** a Department of Neurology, Zydus Hospital, Ahmedabad, Gujarat, India; b Department of Neurology, Ramkrishna Care Hospitals, Raipur, Chhattisgarh, India; c BJ Medical College, Ahmedabad, Gujarat, India; d Department of Pediatrics, Ramkrishna Care Hospitals, Raipur, Chhattisgarh, India; e Department of Hematology, Ramkrishna Care Hospitals, Raipur, Chhattisgarh, India; f Sickle Cell Institute, Raipur, Chhattisgarh; g YLL School of Medicine, National University of Singapore and Division of Neurology, National University Hospital, Singapore.

**Keywords:** cerebral hemodynamics, sickle cell anemia, sickle cell disease, transcranial Doppler

## Abstract

India has the second highest number of cases of sickle cell disease (SCD) and affects the most socioeconomically disadvantaged communities living in a horizontal belt from Gujarat to Odisha state. Despite high prevalence, information about cerebral hemodynamics among children with SCD in India remains scarcely described.

We performed transcranial Doppler (TCD) to assess cerebral hemodynamics among Indian children with SCD and evaluated their association with clinical and hematological parameters.

Children aged 3-18years, diagnosed with SCD living in Raipur in Chhattisgarh and Ahmedabad in Gujarat state were recruited. TCD was performed to obtain flow velocities from middle cerebral (MCA), intracranial internal carotid (ICA) and basilar artery. Associations were evaluated between timed-average-mean-maximum velocities (TAMMV) and various clinical and hematological parameters.

Our prospective study included 62 consecutive children with known SCD. Mean ± SD age of the study population was 9.8 ± 3.9 years and 31 (50%) were male. Mean ± SD hemoglobin was 8.64 ± 1.34 Gm/dL while the mean HbSS ± SD was 70.25 ± 15.27%. While 6 (9.6%) children had suffered from stroke during previous 2 years, 7 (11%) demonstrated abnormal TAMMV. Higher HbSS level along with history of iron chelation therapy, blood transfusion and/or stroke showed a trend towards having higher TAMMV.

Stroke and cerebral hemodynamic alterations are common among Indian children with SCD. Larger studies with detailed neuroimaging and genetic evaluations are needed for better understanding, characterization, risk stratification as well as optimization of the timing of blood transfusion to reduce physical disabilities among Indian children with SCD.

## 1. Introduction

The global burden of Sickle cell disease (SCD) is increasing. The annual number of new-borns with SCD is expected to be more than 400,000 by 2050.^[1]^ SCD results from the inheritance of 2 copies of the sickle β-globin gene variant, affecting hemoglobin.^[2]^ The term sickle cell trait is used for the occurrence of 1 allele coding for hemoglobin with a point mutation on chromosome 11 (HbS), which misinterprets glutamate as valine, and predisposes to clumping of hemoglobin chains and sickling of the red blood cells (RBCs) in dehydrated and deoxygenated conditions. The occurrence of 2 HbS mutation (HbSS) is called SCD and often presents with severe manifestations.^[2,3]^

India is considered to be the second worst affected country, with more than 42,000 babies born with SCD in 2010.^[1]^ Although, HbS trait is widely prevalent in India,^[1]^ exact prevalence of SCD remains only an estimate based on smaller published studies. Previous studies suggest that about 50% of all homozygotes for HbS are born in the states of Karnataka, Tamil Nadu and Maharashtra.^[2]^ In 2016, Rees and Brouse reported that an estimated 120,000 people in India were living with SCD and an equal number were born with the HbS-β-thalassemia genotype.^[4]^

Ironically, there is no estimate available for the number of SCD patients and carriers in Chhattisgarh. A screening project by Chhattisgarh government measured prevalence of SCD among school-age children in its 6 districts between October 2007 and December 2017. Using these screening data, the estimated prevalence of SCD among school-age children was 27,101 while the carriers numbered 714,483.^[5]^ A large-scale population screening for the sickle cell gene screened 359,823 children, aged 3-15 years, in the villages in Raipur District, Chhattisgarh State, India between October 2007 and June 2010. While sickle cell trait was observed in 9.3% children, an SS phenotype was reported only in 0.2%, the low prevalence for the latter being attributed to severe sickness and early death.^[6]^ Importantly, majority of families housing the Beta S allele were observed to be in the lower socioeconomic strata, probably related to the higher prevalence of consanguineous marriages.^[7]^

Individuals with SCD experience considerable morbidity from both acute and chronic sequelae. Without effective treatment, the most severe cases can be fatal within the first few years of life.^[2]^ Various presentations of SCD include chronic hemolytic anemia, recurrent bouts of pain, and organ infarction, including acute ischemic stroke (AIS), hemorrhagic stroke or silent cerebral infarctions at gray-white junctions due to metabolic and oxidative stress.^[8]^

Cerebral infarctions in SCD occur due to the occlusive disease of the distal intracranial segments of the internal carotid artery (ICA), proximal middle (MCA) and anterior cerebral arteries (ACA). Transcranial Doppler (TCD) can detect and monitor intracranial large-vessel disease in patients with SCD. It is portable, safe, noninvasive, easy, relatively low in cost, well tolerated by children and can be performed at bedside or outpatient settings.^[9]^ TCD measures flow velocities in various large intracranial vessels of the circle of Willis reliably and the findings are reproducible. TCD detects a focal stenosis in an intracranial artery by the focally increased velocity in a particular vessel.^[10]^ The role of TCD in stroke prevention among children with SCD was established in a clinical trial by Robert Adams in 1990s.^[11]^ The trial used TCD derived time averaged mean of the maximum velocity (TAMMV) of >200 cm/s as abnormal, which warranted blood transfusion. TAMMV between 170 and 200 cm/s was labeled as “conditional” and a close follow-up was advised while SCD children with TAMMFV <170 cm/s were considered “normal” and they qualified for yearly follow-up.

Although SCD is widely prevalent in various localized belts in India, information about cerebral hemodynamics among the Indian variant is largely lacking. In this prospective study, we aimed to evaluate TAMMV among children living in 2 SCD belts in Gujarat and Chhattisgarh states and examined their association with clinical and hematological parameters.

## 2. Method

This prospective study was conducted at 2 centers: 1 at Zydus ospital Thaltej, Ahmedabad in Gujarat state, and 1 at Ramkrishna Care Hospitals Raipur in Chhattisgarh state. Children of both genders with a confirmed diagnosis of HbSS and aged 3–18 years were included after obtaining an informed written consent by their parents. Patients with ongoing febrile illness, younger than 3 years, suffering from other hemoglobinopathies like HbS Beta thalassemia were excluded.

Data were collected for age, gender, age at first diagnosis of SCD, height, weight, head circumference, history of iron chelation therapy, history of blood transfusion, history of stroke, and current oral iron therapy. Hemoglobin, HbSS, and serum ferritin level were measured for all study participants.

**Figure 1.**
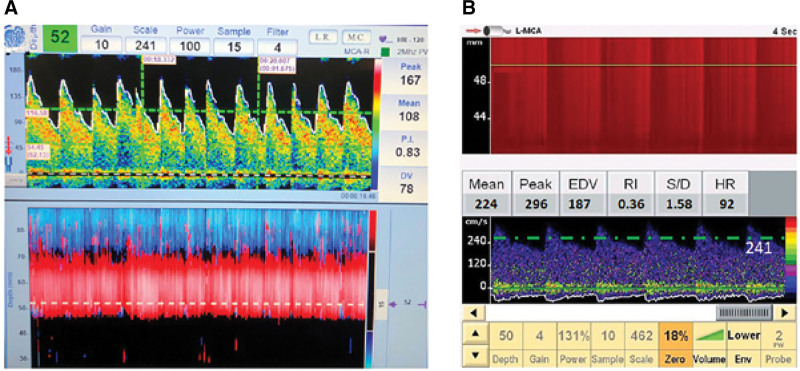
TCD findings from 2 patients from our study. Panel A shows the TCD findings from patent 37. Upper panel shows the Doppler spectra obtained from right middle cerebral artery (MCA), while the lower panel shows the M-mode appearance from RIMED-Digi-lite TCD machine. Note the normal peak systolic velocity, mean flow velocity, and TAMM (116 cm/s), represented by the green dotted line. Panel B shows the TCD findings from left MCA using SONARA-TEK (Carefusion) machine from patient 43. Note the elevated peak systolic velocity, end-diastolic velocity (EDV) and mean flow velocity. Note the calculated time-average mean maximum (TAMM) velocity of 241 cm/s, qualifying for the “abnormal” category.

The procedure and safety of TCD was explained in detail to the study participants and their parents. The head circumference was measured for every child, and TCD was performed using a 3-vessel protocol. Accordingly, intracranial ICA and MCA of both sides were evaluated using the transtemporal acoustic window followed by the evaluation of the basilar artery (BA) through the transforaminal approach. We used Digilite dual channel TCD system (Rimed Inc) and Sonara Tek (Carefusion Inc) with the standard 2-MHz diagnostic probe. Recorded TCD parameters from each intracranial artery included peak systolic velocity (PSV), end-diastolic velocity (EDV). Mean flow velocity (MFV) in cm/s was calculated as EDV + 1/3 (PSV-EDV) for calculating the pulsatility index (PI) as PSV-EDV/MFV. The envelope outlining the spectral waveform through 3 to 5 cardiac cycles was traced electronically and TAMMV was calculated according to the STOP trial as area under the curve (Fig. 1).^[11]^ The study was approved by the institutional ethical committee (2018/zydushospitals/1807).

### 2.1. Statistical analysis

Study data were doubly entered in patient charts and Microsoft Excel 2019. Descriptive statistics were expressed as means (with SD), or counts, as appropriate. For preliminary bivariate analysis, categorical data were assessed using chi square test while continuous variables were analyzed by scatter plots and correlations. A multiple linear regression model was used to determine the independent predictors of TAMMV for MCAs and intracranial ICAs. The Stata statistical software version *R* 4.0.2 for Windows was used for analysis. Significance was taken as *P* value of <0.05.

## 3. Results

Between July 2018 and July 2020, 62 consecutive children, previously diagnosed with SCD (30 from Gujarat and 32 from Chhattisgarh) were recruited. Mean age ± SD of the study population was 9.8 ± 3.9 years and there were 31 (50%) male. All children were in the healthy body-weight category and had low hemoglobin. The mean ± SD age at the time of first diagnosis of SCD was 3.5 ± 2.8 years. Mean hemoglobin ± SD of the study population was 8.64 ± 1.34 Gm/dL while the mean ± SD HbSS was 70.25 ± 15.27%. Baseline characteristics of the study population are represented in Table 1.

**Table 1:** Baseline characteristics of the study population (n = 62).

Parameter	Mean (SD)
Mean age in years (SD)	9.8 (3.9)
Mean age in years at fist diagnosis of SCD (SD)	3.5 (2.8)
Mean head circumference in cm (SD)	50.2 (2.8)
Mean height in meters (SD)	1.24 (0.21)
Mean weight in Kg (SD)	25.5 (10.5)
Mean BMI in Kg/m^2^	16.47 (5.25)
Mean hemoglobin in Gm/dL (SD)	8.64 (1.34)
Mean HbSS in % (SD)	70.25 (15.27)
Mean serum ferritin in mcg/dL (SD)	547.29 (71.29)
Qualitative parameters	
Female gender (%)	31 (50)
History of Iron chelation therapy (%)	21 (33.9)
History of blood transfusion (%)	44 (70.9)
Current oral iron therapy (%)	62 (100%)
History of stroke	6 (9.7)

Abbreviations: BMI = body mass index; HbSS = Hemoglobin SS; SCD = sickle cell disease; SD = standard deviation.

### 3.1. TCD findings

Using the STOP trial criteria, 55 (88.7%) of our study participants demonstrated normal TAMMV. Seven (11.3%) patients had abnormal TAMMV while no one fulfilled the criteria for “conditional” TAMMV. Representative TCD findings from 2 patients are presented in Figure 1.

**Figure 2.**
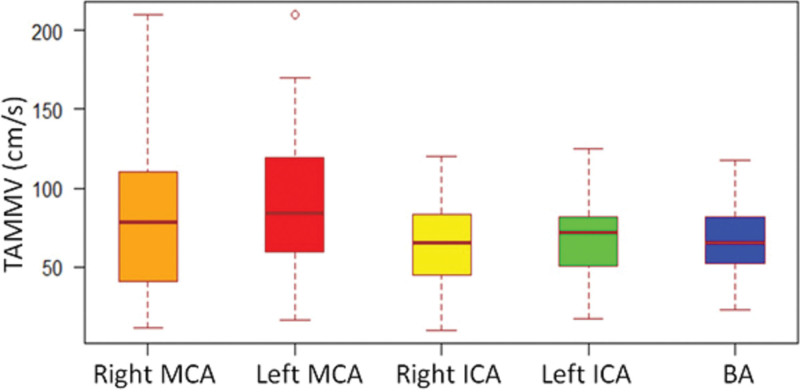
Comparative time-average mean maximum velocities (TAMMV) on transcranial Doppler in various intracranial arteries.

**Figure 3.**
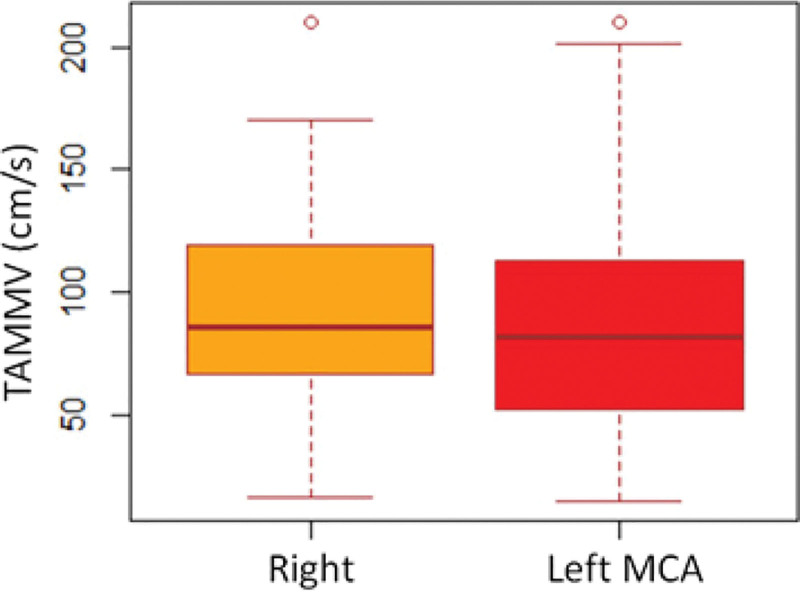
Comparative time-average mean maximum velocities (TAMMV) on the 2 sides.

Table 2 shows the TAMMVs for intracranial ICA and MCA and their relationship with gender, history of blood transfusion, chelation therapy and stroke during previous 2 years. Briefly, TAMMVs were similar in both genders. Although, TAMMV in intracranial ICA and MCA were higher in patients with history of blood transfusion as compared to those without, the difference was statistically significant only for Right ICA [68.7 (28.3) cm/s versus 55.5 (18.8) cm/s; *P* = 0.044]. TAMMV were also significantly higher in patients with history of iron chelation therapy than among those without such therapy [Right MCA 110.9 (61.3) cm/s versus 70.1 (43.1); *P* = 0.006 for Right ICA 74.2 (26.4) versus 59.3 (25.1); *P*=0.042]. No significant differences were observed between TAMMV among patients with history of stroke when compared to those without. Comparative TAMMV for individual intracranial arteries that were evaluated with TCD are shown in Figure 2. Overall, the mean ± SD of the TAMMV did not differ between the 2 sides (left 95.8 ± 43.3 cm/s and right 92.2 ± 51.8 cm/s; *P* = 0.679) as shown in Figure 3.

**Table 2:** Bivariate analysis of timed-average mean maximum velocity (TAMMV) with various confounders (n = 62).

Patient characteristic	Mean (SD) TAMMV (cm/s)			
	Left MCA	Right MCA	Left ICA	Right ICA
Gender				
Male	86 (0.4)	87 (54.3)	69.9 (22.7)	63.8 (25.2)
Female	98.2 (45.6)	86.5 (56.6)	67.7 (28.9)	65.7 (27.8)
*P* value	0.304	0.919	0.750	0.783
Transfusion history				
Yes	93.4 (50.8)	91.5 (58.7)	69.6 (25.6)	68.7 (28.3)
No	89.1(31.5)	76.9 (44.2)	67.1 (27.3)	55.5 (18.8)
*P* value	0.693	0.295	0.752	**0.044**
Iron chelation				
Yes	102.3 (53.8)	110.9 (61.3)	66.8 (27.8)	74.2 (26.4)
No	84.8 (38.3)	70.1 (43.1)	70.1 (24.9)	59.3 (25.1)
*P* value	0.164	**0.006**	0.654	**0.042**
History of stroke				
Yes	100.2 (74.2)	95.7 (49.7)	63.0 (27.8)	64.3 (26.1)
No	91.6 (44.2)	86.7 (55.7)	69.1 (26.1)	64.9 (26.6)
*P* value	0.832	0.749	0.744	0.974

Statistically significant values are highlighted in bold.

Collinearity among the variables was assessed using a matrix scatter plot in which only weight and height showed possibility of hampering the results of our regression model. Therefore, they were removed and body mass index (BMI) was used in their place. A multiple linear regression model was used to determine the independent predictors of TAMMV for MCAs and intracranial ICAs (Table 3). Briefly, age at the time of diagnosis of SCD (HR –8.319, CI –12.795 to –3.844; *P* = 0.0004 for left MCA and HR –8.203, CI –13.740 to –2.667; *P* = 0.004 for right MCA) and head circumference (HR –3.042, CI –5.920 to –0.165; *P* = 0.038 for left ICA and HR –3.002, CI –5.990 to –0.013; *P* = 0.049 for right ICA) showed an inverse relationship with TAMMV, whereas female gender (HR 21889, CI 0.002 to 43.77; *P* = 0.049 only for left MCA) was significantly associated with higher TAMMV. Furthermore, higher HbSS, a positive history of iron chelation therapy, positive history of blood transfusion and stroke were associated with higher TAMM velocities. However, they were not statistically significant.

**Table 3:** Multiple linear regression model coefficients (hazard ratio; 95% confidence interval).

Patient characteristic	TAMM velocities cm/s (95%CI)			
	Left MCA	Right MCA	Left ICA	Right ICA
Age	0.271(–3.431 to 3.973)	–0.256 (–4.836 to 4.324)	–0.606 (–3.073 to 1.861)	–0.866 (–3.428 to 1.696)
*P* value	0.8839	0.911	0.624	0.500
Age at presentation	–8.319 (–12.795 to –3.844)	–8.203 (–13.740 to –2.667)	–1.660 (–4.642 to 1.321)	–1.471 (–4.568 to 1.626)
*P* value	**0.0004**	**0.004**	0.269	0.344
Head circumference	–0.834(–5.153 to 3.485)	–0.88(–6.233 to 4.453)	–3.042 (–5.920 to –0.165)	–3.002(–5.990 to –0.013)
*P* value	0.699	0.739	**0.038**	**0.049**
Hemoglobin	3.826 (–15.216 to –22.869)	2.185 (–21.372 to 25.744)	–2.497 (–15.185 to 10.190)	–3.792 (–16.969 to 9.385)
*P* value	0.688	0.852	0.694	0.565
Hbss	0.228 (–22.432 to 22.889)	1.842 (–26.191 to 29.876)	7.3917 (–7.706 to 22.489)	5.871 (–9.809 to 21.551)
*P* value	0.983	0.895	0.330	0.455
BMI	–2.083 (–4.456 to 0.289)	0.943 (–1.992 to3.878)	–0.536 (–2.118 to 1.044)	0.023 (–1.619 to 1.664)
*P* value	0.0839	0.521	0.498	0.978
Male	1	0	1	1
Female	21.889 (0.002 to 43.77)	12.049 (–15.027 to 39.125)	3.709 (–10.872 to 18.291)	7.467 (–7.678 to 22.611)
*P* value	**0.049**	0.375	0.611	0.327
Transfusion history +ve	–15.746 (–43.890 to 12.397)	–14.346 (–49.164 to 20.470)	6.397 (–12.353 to 25.148)	–1.048 (–20.523 to 18.426)
Transfusion history –ve	1	1	1	1
*P* value	0.266	0.411	0.496	0.914
Iron chelation done	13.968 (–7.492 to 35.429)	43.72 (17.179 to 70.279)	7.817 (–6.481 to 22.116)	–5.866 (–20.716 to 8.984)
No iron chelation	1	1	1	1
*P* value	0.197	**0.001**	0.277	0.431
History of stroke	30.345 (–7.492 to 35.429)	40.096 (–4.686 to 84.879)	9.249 (–14.868 to 33.367)	12.230 (–12.818 to 37.278)
No history of stroke	1	1	1	1
*P* value	0.0985	0.078	0.445	0.331

Statistically significant values are highlighted in bold.

Abbreviations: BMI = body mass index; HbSS = Hemoglobin SS; ICA = intracranial internal carotid artery; MCA = middle cerebral artery.

Scatterplots were created for each vessel’s variables and TAMMV, and on finding a monotonic relationship, spearman correlation rather than Pearson correlation was used to better fit the data as recorded in Table 4. We observed a positive correlation between TAMMV, EDV, and PSV (*P* < 0.001 for all vessels). All correlations were well above 0.7, with PSV having the strongest correlation with TAMMV (more than 0.9 in all vessels). Pulsatility index also showed a strong correlation with TAMMV in all vessels, albeit weaker than with other parameters.

**Table 4:** Spearman correlations between cerebrovascular parameters of transcranial Doppler (TCD).

Other cerebrovascular TCD parameters	TAMM velocities (cm/s)				
	Left MCA	Right MCA	Left ICA	Right ICA	BA
End diastolic velocity	0.818	0.866	0.705	0.781	0.746
*P* value	**<0.001**	**<0.001**	**<0.001**	**<0.001**	**<0.001**
Peak systolic velocity	0.928	0.966	0.945	0.952	0.947
*P* value	**<0.001**	**<0.001**	**<0.001**	**<0.001**	**<0.001**
Pulsatility index	0.699	0.508	0.339	0.307	0.445
*P* value	**<0.001**	**<0.001**	**0.009**	**0.020**	**<0.001**
Mean flow velocity	0.942	0.941	0.794	0.861	0.816
*P* value	**<0.001**	**<0.001**	**<0.001**	**<0.001**	**<0.001**

Statistically significant values are highlighted in bold.

Abbreviations: ICA = intracranial internal carotid artery; MCA = middle cerebral artery; TAMM = timed averaged mean maximum velocity.

## 4. Discussion

Our study provides important information about cerebral hemodynamic parameters and their relationship with various confounding factors for Indian patients with SCD. To the best of our knowledge, this is the first study to provide such data.

TCD is an indispensable tool for the evaluation of patients with SCD and preventing stroke by guiding the appropriate timing of blood transfusion.^[12–14]^ Compared to other imaging techniques, TCD is preferred for being noninvasive, cost effective, and for its ability for performance in an ambulatory setting.^[9,11–13,15]^ Therefore, TCD is perhaps, the most appropriate tool for countries with limited resources.

Initially, we employed a 2-vessel TCD protocol, which eliminates evaluation of the posterior circulation. This approach enables a rapid test performance even by relatively inexperienced sonographers, especially since BA insonation requires specialized skills for children whose heads are not often stationary during the test. However, once satisfactory Doppler spectra were obtained from MCA and intracranial ICAs, we extended the evaluation to BA in all study participants. Interestingly, BA flow characteristics showed the correlations similar to MCA and intracranial ICA.^[16]^

About 11% of our study participants showed TAMMV of more than 200 cm/s. This proportion in an unselected population of patient with SCD was higher than the TAMMV reported from Africa,^[17]^ Brazil,^[18]^ and Mali.^[19]^ This emphasizes that SCD is a significant problem in India, which remains poorly studied and less reported. We feel that there may be many other unexplored contributing factors and differences in disease epidemiology in Indian variant of SCD, like genetic (G-6PD deficiency, Beta-chain haplotypes, and alpha thalassemia like hemoglobinopathies) and environmental (climate, seasonality, vitamin, and mineral intake and nutritional status).^[20,21]^

We observed significant associations of high TAMMV with lower age of presentation. This observation is consistent with many previous reports.^[17,19,21,22]^ Although, age was inversely associated with TAMMV, when data were adjusted for height, weight and BMI, this association was not found to be statistically significant. Interestingly, head circumference showed a significant inverse relationship with TAMMV, which is difficult to explain.

Anemia and the resultant hypoxia in SCD are believed to cause chronic cerebral vasodilation and elevated cerebral blood flow and velocities.^[11,12,16]^ Accordingly, blood Hb and HbS levels may be considered as indirect measures of the severity of anemia and cerebral hypoxia. However, contrary to the existing literature, we did not find an association between HbS and TAMMV.^[22,23]^ We believe that this observation could be a statistical error due to the small sample size of our study. Our belief is supported by the inverse association of TAMMV with history of blood transfusion in recent past. Interestingly, history of stroke also showed an association with higher TAMMV. These findings are consistent with previous studies and lend support for the recommendation of performing frequent TCD evaluation for Indian patient with SCD and blood transfusion for selected patient according to the STOP trial protocol.

Instead of using the STOP trial criteria of TAMMV, some studies used PSV for determining the timing of blood transfusion, in order to avoid the nuances of calculating the former. Accordingly, PSV of >250 cm/s was considered “abnormal” while the PSV values between 200 and 250 were labeled as “conditional”.^[24–26]^ Interestingly, in our study, PSV provided highest correlations (>0.9). However, using PSV cutoff values, only 4 patients could be classified as having “abnormal” PSV as compared to 7 patients who qualified for being “abnormal” by the standard TAMMV criteria used in the pivotal STOP trial.^[11]^ Our observations could have been biased due to the small sample size or other factors like milder disease in Indian SCD patients, as their Beta-globin mutation is on chromosomes with Saudi Arabia/India beta gene haplotype.^[27,28]^

## 5. Limitations

We acknowledge some limitations of our study. First, the small sample size limits our ability to draw definite conclusions. *Furthermore, potential biases by the different limiting factors could not be explored due to the smaller sample size.* Second, only few patients in our study cohort suffered from stroke in recent past. Perhaps, this might explain why the Indian SCD is a considered a milder variant due to differences in the genetic mutations and therefore, remains less reported. Third, we did not perform magnetic resonance imaging or computerized tomography of the brain in our patients, which could have shown a higher proportion of “silent” cerebral infarcts and allowed us to establish better correlations with cerebral hemodynamic parameters. Despite these limitations, our manuscript provides compelling information for performing larger well-designed comprehensive studies on SCD, which usually affects underprivileged sections of the society.

## 6. Conclusions

Our pilot study shows that considerable proportion of patients with Indian SCD suffer from various alterations in cerebral hemodynamic parameters. We recommend larger studies with detailed neuroimaging and genetic evaluations for better understanding and characterizing the Indian variant of SCD. Such studies would help in optimizing various therapeutic approaches and limit the physical disabilities among children living in SCD belts with poor socio-economic resources.

## Author contributions

Conceptualization: Bhakti Gajjar, Sanjay Sharma, Arvind K. Sharma

Data curation: Bhakti Gajjar.

Formal analysis: Bhakti Gajjar, Vijay K Sharma

Investigation: Bhakti Gajjar, Sanjay Sharma, Erum Khan, Pranita Sharma, Pawan Jain, Vikas Goel, Arvind Neral, Jyotish Patel, Mamta Parmar, Kanika Sharma, Vijay K Sharma, Arvind K Sharma.

Methodology: Bhakti Gajjar, Sanjay Sharma, Arvind K Sharma, Vijay K Sharma.

Supervision: Sanjay Sharma, Arvind K Sharma.

Validation: Arvind K Sharma, Sanjay Sharma, Vijay K Sharma.

Visualization: Arvind K Sharma, Sanjay Sharma, Vijay K Sharma.

Writing – original draft: Bhakti Gajjar, Erum Khan

Writing – review & editing: Bhakti Gajjar, Sanjay Sharma, Erum Khan, Pranita Sharma, Pawan Jain, Vikas Goel, Arvind Neral, Jyotish Patel, Mamta Parmar, Kanika Sharma, Vijay K Sharma, Arvind K Sharma.
